# Acknowledgement to Reviewers of *Medicina* in 2018

**DOI:** 10.3390/medicina55010022

**Published:** 2019-01-20

**Authors:** Medicina Editorial Office

**Affiliations:** MDPI, St. Alban-Anlage 66, 4052 Basel, Switzerland

Rigorous peer review is the cornerstone of high-quality academic publishing. The editorial team greatly appreciates the reviewers who contributed their knowledge and expertise to the journal’s editorial process over the past 12 months. In 2018, a total of 112 papers were published in the journal, with a median time to first decision of 17 days and a median time to publication of 51.5 days. The editors would like to express their sincere gratitude to the following reviewers for their cooperation and dedication in 2018:
Abuzaid, AhmedLing Choo, PeiAlessandri, ClaudiaLlaneza-Suarez, DavidAl-Kindi, SadeerLo, Shyh-ChyiAllegra, EugeniaLoffredo, LorenzoAlsharairi, NaserLópez-López, DanielAnadol, RemziLu, Da-WenAnderson, RobertLudy, Mary-JonAnsa, BenjaminLuft, Friedrich C.Anyfantakis, Dimitrios I.Lukaski, Henry C.Archer, DavidMagan-Fernandez, AntonioArmando Gagliardi, PaoloMalek, NaveedArnold, Robert W.Manjunath, ManuboluAshish, PathakMarci, RobertoAssari, ShervinMascherini, GabrieleAterini, StefanoMasulli, MariaAubert, RachaelMichels, Alexander J.Azaiez, HelaMiederer, MatthiasBaba, YuhMishima, MasanoriBäck, MagnusMistiaen, WilhelmBasak, DebasishMitsiakos, GeorgiosBathula, ChandraMorales, María M.Beeken, Rebecca J.Moschini, ElisaBenesic, AndreasMountjoy, KathyBidarimath, MallikarjunMukwevho, EmmanuelBogomolnaya, Lydia M.Murovska, ModraBrady, Patrick G.Musk, BillBricard, DamienMuthukumar, ThangaveluBurgon, Patrick G.Nanba, KazutakaBužgová, RadkaNasser, MohdCaron, KathleenNeogi, UjjwalCarroll, Kevin M.Nijman, Ruud G.Celia, ChristianNorihiko, NaritaChacón Cuberos, RamónNowak, Michal S.Che, MeixiaOxford, Samuel W.Cheng, I-ShiungPace, NelsonChesnaye, Nicholas C.Pagano, StefanoChiang, Hsiu-MeiPappas, Richard S.Chiou, Chien-ShunPawar, KasturiCimmino, GiovanniPérez-Badia, RosaColey, Helen M.Pérez-Sayáns, MarioCortés Castell, ErnestoPerrier, JosetteCzłonkowska, AnnaPhilpott, Carl M.Daniels-Wells, Tracy R.Pitteri, MarcoDas, AnshumanPlaza-Díaz, JulioDavid, Michael Z.Poulton, Alison S.De Morís, CésarPoulton, Alison SallyDe Toni, LucaPowers, James S.Degn Vinther, MatildaPuertas Molero, PilarDevy, JérômeQuevedo, LluisaDi Stasio, DarioQvarnstrom, YvonneDiamantis, PlachourasRago, AnnaDimopoulos, KonstantinosRamachandiran, IyappanDolowy, MalgorzataRamadas, AmuthaDoucette, LanceRaman, DayanidhiDouglas Baker, MarkRathinasabapathy, AnandharajanDuquette, PierreReaman, Gregory H.Durazzo, AlessandraRen, JunDusi, VeronicaRikhtegar, FarhadEldridge, Alison L.Rivera, VictorElmarakby, AhmedRobitaille, EricFabiani, RobertoRobson, Philip M.Fahim, AhmedRocque, Brandon G.Faust, Andrew C.Rodriguez, Erika F.Fechner, HenryRothe, HansjörgFedele, FrancescoRotolo, NicolaFelder, Robin A.Sacconi, LeonardoFerguson, BradleySachpekidis, ChristosFettiplace, Michael RobertSaeedi, PouyaFina, EmanuelaSanchez-Dalmau, Bernardo F.Flack, Kyle D.Sankarasubramanian, VishwanathFourtounas, CostasSantulli, GaetanoFrame, Leigh A.Sarapultsev, AlexeyGajda, MaksymilianSarpong, Eric M.Gamet-Payrastre, LaurenceScarano, AureliaGeorgian, BadicuSchaefer, FranzGiussani, GiorgiaSchwarz, Kathleen B.Górska, SabinaSedlak, LechGrammatiki, MariaSeijas, RobertoGray, AndrewSessa, AurelioGregg, LucileSilveira-Moriyama, LauraGreve, Anders M.Skeberdis, Vytenis ArvydasGrjibovski, AndrejSmallfield, George B.Gupta, VijayalaxmiSmaoui, SlimGupta-Rossi, NeetuSohn, JutaeH. J., VictoriaStankevičius, EdgarasHammer, SuntreaStanley, Jone A.Han, DongStefanoff, PawelHans, GuyStevens, DavidHarambat, JeromeSukumaran, AnilHermida Prieto, ManuelSun, QianHerwadkar, AnushreeSun, ZhonghuaHetta, HelalSzabó, AndrásHill, EthanTagawa, TsuyoshiHimoto, TakashiTarkkila, PekkaHoefsloot, E.H.Teotia, PoojaHooper, Douglas C.Thomas, Seddon Y.Horwitz, HenrikTiso, NatasciaHsieh, Yi-ChenTorres Lagares, DanielHwan Kwak, JongTsai, Feng-ChunIde, KazukiTsai, JamesIlmer, MatthiasTseng, Tung-SungIvanov, Alexander V.Tsukiyama-Kohara, KyokoJaradat, NidalTuells, JoséJelleyman, CharlotteTursan D’Espaignet, EdouardJeong, Keun-YeongVaidyanathan, BharatJuliusson, GunnarVamanu, EmanuelKadambi, Pradeep V.Van Ballegooijen, HanneKatzenstein, David A.Van Empelen, PepijnKaushik, MaheshVan Speybroeck, AlexanderKi Ju, ManVarju, CeciliaKim, Sahn-HoVenturini, CarolaKim, YoonaVoltas, NúriaKocot, JoannaWade, Charles E.Kohga, AtsushiWanat, MartaKoppe, Janna G.Wardenaar, FlorisKorfei, MartinaWei, ChangyongKosari, SamWeyer, AndyKosinski, PawelWilliams, Mark E.Kriegel, AlisonWilliams, Steven E.Kubatka, PeterWu, RongxueKumar, NitinXin, MeiKumar, SunilYanagisawa, SatoshiKuwabara, TakashigeYeo, SynLang, LiweiYiin, Lih-MingLavender, AndrewYui Kwan, MariaLavtar, PolonaYuzawa, YukioLaycock, HelenZaffagnini, StefanoLeijs, Marike M.Zhang, ShanshanLi, Shengwen CalvinZhao, BaoyinLiao, Jyh-feiZurita Ortega, Félix

